# Radical Cystectomy is the best choice for most patients with muscle-invasive bladder cancer? Opinion: Yes

**DOI:** 10.1590/S1677-5538.IBJU.2017.02.03

**Published:** 2017

**Authors:** Leonardo L. Monteiro, Wassim Kassouf

**Affiliations:** 1 Division of Urology, McGill University Health Center, Montreal, Quebec, Canada

**Keywords:** Urinary Bladder Neoplasms, Cystectomy, Chemoradiotherapy, Adjuvant, Neoadjuvant Therapy

Around the World, radical cystectomy (RC) with bilateral pelvic lymphadenectomy (PLND) and perioperative chemotherapy is regarded as the standard treatment for patients with muscle-invasive bladder cancer (MIBC). This management approach is supported by numerous renowned organizations and guidelines, such as the National Comprehensive Cancer Network (NCCN), as well as by the European Association of Urology (EAU) guidelines. In fact, the latter has assigned RC a grade A recommendation for treating MIBC (T2-T4aN0M0) and high-risk non-muscle invasive bladder cancer.

Furthermore, several studies demonstrate that quality surgery with RC and PLND offers excellent locoregional control for patients with clinically localized or locally advanced bladder cancer, even at extended long-term follow-up (1, 2). Therefore, RC accomplishes a paramount target in bladder cancer. It achieves oncologic control with acceptable morbidity. However, in the modern World, oncologic control is not the only goal when physicians decide to treat cancer patients, as quality of life is very important as well. In the absence of prospective randomized studies, it is not possible to compare RC versus other forms of therapy. There would be a strong bias because patients are subjected prior to therapy for selection of a specific treatment approach and hence patients are prepared for the disadvantages and advantages associated with the different treatment approaches. As such, there is a real risk that patient-led preferences would be inappropriately labeled against RC.

Even though there can be considerable complication rates, we believe that RC remains the preferred choice for most patients with MIBC. To support this reasoning, we ask ourselves one simple question: Is there some other treatment with equivalent or better oncological outcomes than RC?

Organ-preserving therapies have been developed as an alternative to radical treatments for organ-localized cancer disease. The purpose of organ-preserving therapies is to improve patients’ quality of life and to reduce the associated morbidity of radical treatments, while maintaining similar oncologic outcomes. For instance, head and neck, breast, anal, and prostate cancers have well-established organ-preserving therapies. In patients with MIBC, there are also several bladder preservation options, such as single modality treatments that include transurethral resection of a bladder tumor (TURBT), radiation therapy (RT), or chemotherapy alone, as well as multimodality treatments. Tri-modality therapy (TMT) is currently the most accepted form of multimodality bladder preservation therapy.

Large retrospective non-selected series from single institutions have shown that the recurrence-free survival (RFS) rates following RC ranges from 62% to 68% and 50% to 66% at 5 years and 10 years, respectively (3-5). Moreover, Hautmann et al. published similarly long-term oncologic outcomes among 1,100 patients who underwent RC; they exhibited an overall 10-year cancer-specific survival (CSS) rate of 67% (6).

TMT for MIBC is the most studied and most effective bladder-sparing treatment. TMT consists of maximal TURBT followed by radiotherapy and concomitant radio-sensitizing chemotherapy. It also includes prompt salvage RC in patients with incomplete response or among those who developed invasive recurrence.

In a systematic review, contemporary bladder-preservation modalities were assessed, while focusing specifically on TMT in MIBC (7). This review included 83 studies in its synthesis, which consisted of 5 prospective TMT Phase III trials, and 2 Phase III randomized controlled trials (RTCs). The remaining articles consisted of large retrospective series and Phase II trials that were conducted with small cohorts. Overall, the mean response rate following TMT was 73%. The types of cancers that were optimal and eligible for bladder preservation were those with low-volume T-2 disease without hydronephrosis or extensive carcinoma *in situ* who underwent resection of all visible disease prior to therapy. Following TMT, the rate of recurrent bladder tumors ranged from 24%-43%, and the salvage cystectomy rate was 25-30%. Although feasible, salvage cystectomy after TMT is associated with a slightly higher risk of complications, and almost all patients end up receiving ileal conduit diversion instead of orthotopic neobladder reconstruction. In the same review, bladder preservation approaches with TMT were not found to be free of complications; the acute grade 3-4 toxicity rates ranged from 10%-36%. Most studies with TMT had small cohorts (<50 patients) or limited follow-up data; they also featured heterogeneous treatment protocols that provided few data on long-term oncologic control or late toxicity.

In a recent multicenter randomized trial, oncological outcomes in patients with BC that underwent RT alone or in combination with chemotherapy were assessed. Overall, 83% of patients had clinically organ-confined disease (<cT2), 33% of patients received neoadjuvant chemotherapy and 13% developed late grade 3-4 toxicity. As patients on clinical trials are generally biased towards better outcomes, it is somewhat unexpected, that the 5-year overall survival (OS) rates were only 48% (95% CI: 40-55) in the TMT group and 35% (95% CI: 28-43) in the radiotherapy group (8,9).

Recently, researchers at the Massachusetts General Hospital published the largest single-institution experience of TMT for MIBC, where 475 patients were treated between 1986 and 2013 (10). The patients enrolled in this study presented with the following characteristics: 67% were in the cT2 stage, 88% were without hydronephrosis, and 76% showed an absence of tumor-associated CIS. However, patient selection criteria became more stringent after 2005 where 97% of patients had cT2 disease, 100% were without hydronephrosis, 81% showed absence of CIS. This retrospective analysis where 73% of patients received adjuvant chemotherapy following TMT yielded actuarial 5-year and 10-year CSS rates of 66% and 59% respectively after a median follow-up of 6.9 years among survivors. Moreover, the risk of salvage cystectomy at 5 years was 29%. This study demonstrates that in well-selected patients, TMT offers comparable outcomes. Our center has had a long-standing interest in TMT. We strongly believe that patient selection remains critical to good outcomes. We have reported on our earlier experience when patients were not selected appropriately (e.g. only 10% had complete TURBT), the 5-year CSS was 38% (11). However, our more recent experience with more stringent criteria and properly selected patients yielded significantly improved outcomes with 3-year CSS of 71% (12).

Patient selection improves outcomes even in patients treated with radical cystectomy. Many papers attempt to compare TMT with non-selected surgical series where outcomes can appear similar. However, if one uses similar selection criteria as within the TMT studies, the outcomes of surgical series are excellent. A recent study that clearly illustrates this point was from the University of Texas MD Anderson Cancer Center and the University of Southern California, who treated patients with MIBC (cT2) who are considered low risk (i.e., absence of hydronephrosis, palpable mass, >cT3b disease, or lymphovascular invasion on TURBT), showed an excellent 5-year CSS rate of 83% (13).

Lymphadenectomy has been associated with improved survival rates in multiple surgical studies (14), and the number of lymph nodes removed is an independent predictors of survival (15, 16). This therapeutic benefit is not fully addressed in TMT studies. There are also some concerns to consider complete response on transurethral biopsy after TMT as an excellent marker of good response because there are contrasting studies showing presence of residual tumor after RC in patients labeled complete responders. For example, the Memorial Sloan Kettering Cancer Center experience demonstrated that after methotrexate, vinblastine, doxorubicin and cisplatin (M-VAC), chemotherapy alone, 30% of patients had residual MIBC on cystectomy that was not detected on the preoperative TUR (17).

While one can use arguments to support both therapeutic sides, a comparison between different bladder-preservation modalities and RC remains difficult. Most published studies are largely heterogeneous with regards to the patient sample, tumor staging (clinical versus pathological), treatment modalities, and data reporting and analysis methods. As such, it is difficult to obtain good evidence to settle the debate on whether TMT has equivalent oncological outcomes to RC. A direct comparison between RC and TMT has been confounded by selection biases and discordance between clinical versus pathologic staging. There are no prospective randomized trials to date that have compared TMT with RC and pelvic lymphadenectomy following neoadjuvant chemotherapy. Since the “Selective Bladder Preservation Against Radical Excision” trial failed to recruit patients (18), it is likely that RCTs in this domain will never be completed. Overall, cumulative data have shown that TMT leads to acceptable outcomes and may be considered a reliable alternative option in well-selected patients. However, based on a large body of evidence, RC remains the optimal choice for most patients with MIBC.

While we are presenting the ‘Pro’ side for RC in this manuscript, it is important to highlight that the two approaches (RC or TMT) should be used in a complementary fashion to ensure most patients with MIBC are actually receiving definitive therapy. Recent studies raise concern that 50% of patients with MIBC are not receiving any form of definitive therapy (19, 20). Most patients who are not surgically fit to undergo radical cystectomy will be able to tolerate TMT. Furthermore, even in patients that are not ideal candidates for bladder preservation therapy and who are not surgical candidates, they should still be offered TMT as cure can still be achieved.
